# Intestinal necrosis caused by acute mesenteric ischemia associated with pregnancy: A case report and literature review

**DOI:** 10.1016/j.ijscr.2020.08.009

**Published:** 2020-08-21

**Authors:** Dora Corrales, Daniel A. Giraldo

**Affiliations:** a Departamento Materno-infantil del Hospital III EsSalud - Chimbote, Peru; b Universidad Peruana Cayetano Heredia, Facultad de Medicina - Lima, Peru; c Sociedad Científica de Estudiantes de Medicina Cayetano Heredia - Lima, Peru

**Keywords:** Mesenteric ischaemia, Pregnant women, Inflammatory bowel diseases, Cardiovascular diseases

## Abstract

**Introduction:**

Mesenteric ischaemia associated with pregnancy is a rare disease with a high mortality rate and its prognosis depends on the time passing between diagnosis and final management.

**Presentation of case:**

A 38-year-old Peruvian woman developed intestinal necrosis during week 35 of her pregnancy because of mesenteric ischaemia associated with her pregnancy.

**Discussion:**

Given the findings of extensive intestinal necrosis, surgical resection was performed, causing short bowel syndrome. Histopathological analysis confirmed that the immediate cause of the patient’s ischaemia was venous thrombosis associated with her gestation.

**Conclusion:**

Acute mesenteric ischaemia should be considered as a possible diagnosis in pregnant woman with risk factors for thrombophilia presenting with abdominal pain.

## Highlights

• Acute mesenteric ischemia associated with pregnancy is a rare pathology that is difficult to treat.
• Acute mesenteric ischemia can be caused by a venous thrombosis associated with gestation.
• Acute mesenteric ischemia should be considered in pregnant woman.
• Patient with intense intestinal necrosis, need a intestinal resection which would cause a short bowel syndrome.

## 1 Introduction

Acute mesenteric ischaemia (AMI), commonly defined as a decrease in blood flow that nourishes and oxygenates one’s intestinal tissue, causes lesions in the tissue and subsequently leads to infarction and intestinal necrosis. Stefan Acosta has reported in his study concerning the epidemiology of mesenteric vascular diseases that the overall incidence rate of AMI is 12.9/100,000 patients per year [1]. It is important to know the clinical manifestation of AMI because of three main reasons: First, it is the most common cause of an acute abdomen, more common than appendicitis and rupture of abdominal aortic aneurysm, in patients older than 75 years [2]; second, it has a high mortality rate, of approximately 82% after diagnosis, showing a palpable difficulty in its management [3]; third, it is usually a very difficult pathology to diagnose, which starts showing symptoms only at the beginning of intestinal infarction, and specific diagnostic tests for it are lacking.

We report the case of a 38-year-old pregnant patient with mesenteric ischaemia associated with her pregnancy. During surgery, extensive necrosis was observed.

This work has been reported in accordance with the SCARE criteria [4].

## 2 Presentation of case

A 38-year-old female at 35-week and 6-day gestation presented at the gyno-obstetrics emergency room of our institute early morning with abdominal pain in the epigastrium. The pain suddenly appeared and aggravated over an hour. Her obstetric history was G 4 P 0+3+0+2, and she has a history of gestational complications. In her first pregnancy, placental abruption occurred at 39 weeks, and the neonate (weight, 4900 g) was born dead after a caesarean operation. The second pregnancy had a diagnosis of severe preeclampsia at 37 weeks, and the neonate (weight, 3800 g) was born after performing a caesarean operation. Finally, in her third pregnancy, she had preterm labour at 36 weeks, and the neonate (weight, 3400 g) was born after a caesarean operation.

During physical examination, the patient was hemodynamically stable, and the bowel sounds were verified during abdominal auscultation. She had an evident general malaise characterized by abdominal pain in the epigastrium associated with bilious vomiting. The clinical evaluation showed active gestation without foetal well-being.

After clinical evaluation, the doctor decided to initiate analgesic and antispasmodic treatment (sodium metamizole, 1 g/2 mL + scopolamine butylbromide, 20 mg/mL + ranitidine 50 mg + hydration with 0.9% NaCl × 1 L). Pain persisted in the patient 1 h after pharmacological treatment despite the administration of analgesics. Foetal heartbeats were 138 per min. Therefore, the patient was referred to the Surgery and General Internal Medicine department.

The Surgery department evaluated the pregnant patient and noted evident pain in the epigastrium and right hypochondrium. They suspected biliary colic, and accordingly, requested auxiliary laboratory tests (Table 1) and imaging studies (abdominal ultrasound) to rule out acute biliary conditions.

**Table 1:** Auxiliary laboratory tests.

Hematic Biometrics	RBC: 4.44 × 10^6^/uL, Hb: 11.9 g/dL, PCV: 36.0%, MCV: 79.1 fL, MCH: 26.8, PC: 383 × 10^3^/uL, **WBC: 13.99 × 10^3^/uL**, Nφ: 63.1%, Lym: 26.5%, Mo: 5.2%, Eos: 1.8%, Bas: 0.3%
Liver and Kidney Function Tests	TB: 0.58 mg/dL, DB: 0.10 mg/dL, IB: 0.48 mg/dL, albumin: 3.8 g/dL, globulin: 3.93 g/dL, glucose: 174 mg/dL, creatinine: 0.43 mg/dL, ALP: 121 U/L, ASP: 14 U/L, ALT: 9 U/L, urea: 17 mg/dL
Coagulation and Blood Profile	BT: 2.00 min, CT: 8.00 min, **PT: 42.1 s**, INR: 2.64, blood group: B, RH (+)
Serum electrolytes	pH: 7.18, Na: 141 mmol/L, K: 3.6 mmol/L, Ca: 0.94 mmol/L, Cl: 114 mmol/L, lactate: 5.2 mmol/L, bicarbonate: 14.0 mmol/L, CO: 291.5 mmol/kg

Red blood cell count (RBC), haemoglobin (Hb), Packed cell volume (PCV), Mean Corpuscular Volume (MCV), Mean corpuscular haemoglobin (MCH), Platelet Count (PC), White Blood Cell Count (WBC), Neutrophils (Nφ), Lymphocytes (Lym), Monocytes (Mo), Eosinophils (Eos), Basophils (Bas), Total Bilirubin (TB), Direct Bilirubin (DB), Indirect Bilirubin (IB), Alkaline Phosphatase (ALP), Aspartate aminotransferase (ASP), Alanine aminotransferase (ALT), Bleeding Time (BT), Clotting time (CT), Prothrombin time (PT), International Normalized Ratio (INR), Calculated osmolarity (CO).

The General Internal Medicine department evaluated the patient as she continued to present with severe abdominal pain and suggested optimizing analgesia with the use of opiates. They too suspected biliary colic.

Three hours after admission, the patient continued to experience pain despite the new drug therapy. The abdominal ultrasound report (Table 2) showed probable intestinal involvement associated with foetal bradycardia of 80–90 beats per minute (BPM). Accordingly, the attending gynaecologist decided to admit the patient to the operating room, and obstetric and surgical intervention was performed (Graphic 01).

**Table 2:** The abdominal ultrasound report.

Liver, common bile duct, and gallbladder	Right liver lobe sized 16.8 cm with mild signs of liver disease. Portal vein with appropriate calibre. Free subhepatic fluid observed. No focal lesions, no bile duct dilation. Gallbladder sized 70 × 30 mm, with slight sedimentation and no gallstones.
Pancreas	Not visible from the pregnant abdomen.
Bowel loops	Some slightly dilated intestinal handles with thickened walls observed. Thus, intestinal involvement or other associated aetiology is not ruled out.
Foetus	Foetal cardiac arrhythmia observed with accentuated bradycardia (81–91 bpm). Consider foetal distress.

Obstetric intervention: A median infraumbilical incision was made. Transverse segmental hysterotomy was performed, a female newborn was delivered by a paediatrician. A hysterorrhaphy in 2 planes was made with ligation of both uterine tubes before the surgeons intervened.

**Fig. 1:**
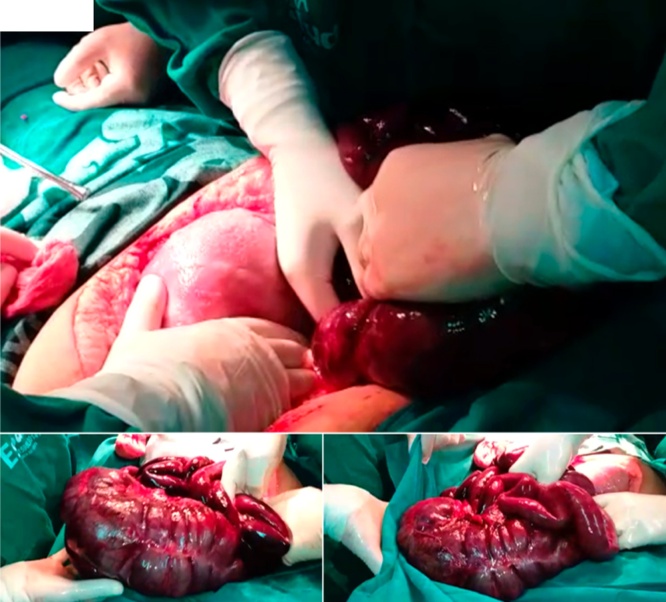
Necrosis found during surgical procedure. Necrosis of intestinal loops of the jejunum, ileum, cecum, ascending colon and proximal 2/3 of the transverse colon.

Surgical intervention: The surgeons detected necrosis of intestinal loops of the jejunum, ileum, cecum, ascending colon, and proximal 2/3 of the transverse colon (Fig. 1). Surgical resection of the entire necrotized intestinal segment was decided. Side-to-side anastomosis between the duodenum and the transverse colon was performed, and subsequently, the cavity was washed with heated physiological serum. Finally, points were placed at the meso-level of the jejunum to close the anastomotic gap and the wall was sutured (Fig. 1A, B, C).

Postoperatively, the patient was moved to the Intensive Care Unit for normalization of homeostasis, post-sepsis stabilization, and compulsory parenteral nutrition. The patient recovered successfully; a central venous catheter was maintained for nutritional support. The patient was discharged from the hospital and transferred to the Short Intestine Unit of a specialized hospital in Lima, Peru.

## 3 Discussion

Mesenteric ischaemia is a rare disease, with non-specific early clinical presentation and rapid development. The condition may aggravate in a short duration and may even lead to death caused by sepsis, multiorgan failure, and shock [5].

The occurrence of AMI in association with pregnancy is very rare. However, this association may be attributed to the physiology of pregnancy itself, where there is an obvious increase in factors VII and VIII-C and fibrinogen, which cause a hypercoagulable state [6,7]. All of these changes prepare the body to cope with blood loss during childbirth, but also increase the risk of thromboembolism [7,8].

Furthermore, the diagnosis in this case had different limitations. Generally, tomography and contrast angiography are the most effective diagnostic imaging tools for AMI [[9], [10], [11]]. However, given the pregnancy condition, the possibility of using tomography was ruled out. Moreover, contrast angiography is too invasive and therefore cannot be considered in all pregnant patients [12]. Furthermore, in our case, the hospital did not have angiography equipment available.

With respect to management, anticoagulant and thrombolytic therapies can be considered in patients without necrosis, but in the case of intestinal necrosis, bowel resection should be performed [13,14]. In our patient, conservative vascular management, such as performing embolectomy of the visceral vessels, was not viable because of evidence of intestinal necrosis and a systemic inflammatory response [14]. Given the findings of intestinal necrosis, a resection was performed, which caused short bowel syndrome but saved the immediate life of the mother and the premature newborn.

Finally, the aetiology of the mesenteric ischaemic disease can be of arterial or venous origin. Statistically, it has been found in autopsies that acute arterial occlusion of the mesenteric artery is more frequent (incidence rate, 68%) compared with mesenteric venous thrombosis (incidence rate, 16%) [1]. Cases of arterial origin are generally associated with elderly patients, with chronic diseases, dyslipidaemia, hypertension, and a history of acute myocardial infarction, which cause a thrombus or embolus that shoots towards the mesenteric artery and occludes the vessel [1,2]. In contrast, mesenteric venous thrombosis is associated with thrombophilia, with a greater occurrence in women of reproductive age with a history of repeated abortions, coagulopathies, or autoimmune disease disorders [1,7,15]. Placental abruption is also known to be associated with thrombophilia [7].

In the laboratory examinations of the patient (Table 1) the most striking values were the following: white blood cell count of 13.99 × 10^3^/μL (normal range: 4.5–11.0 × 10^3^/μL) and prothrombin time (PT) of 42.1 s (normal range: 11–13.5 s). Elevated PT suggests an alteration of the extrinsic pathway of coagulation and hence coagulopathies involving these factors [16].

**Fig. 2:**
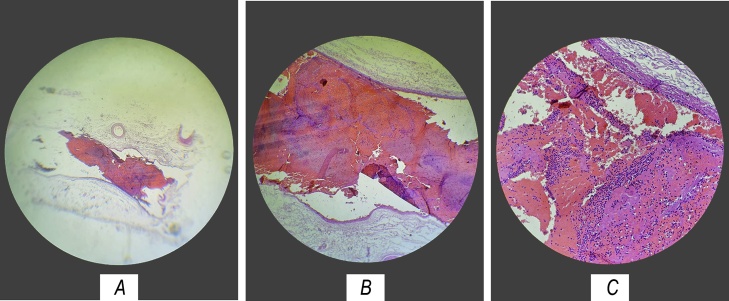
Histopathological imaging. Presence of thrombi in a mesenteric venous vessel.

The histopathological report showed evidence of ischaemic haemorrhagic necrosis extending to the endothelium, subendothelium, and part of the smooth muscle. In addition, mesenteric venous vessels with the presence of thrombi were visualized, which confirmed that the immediate cause of the patient’s ischaemia was a venous thrombosis associated with her pregnancy (Fig. 2A, B, C).

## 4 Conclusion

It is important to maintain a diagnostic suspicion of AMI in cases of abdominal pain in pregnant women with risk factors for thrombophilia. Analysis and imaging studies should be performed as soon as possible to improve patient prognosis.

## Declaration of Competing Interest

The authors report no declarations of interest.

## Sources of funding

No source of funding to be declared.

## Ethical approval

This case report was conducted with the approval of the Medical Ethics Committee of the Hospital III – Essalud.

## Consent

Written informed consent was obtained from the patient for publication of this case report and accompanying images. A copy of the written consent is available for review by the Editor-in-Chief of this journal on request.

## Author contribution

**Corrales Dora:** Conceptualization, Methodology, Writing- Reviewing and Editing, Investigation, Resources, Validation, Supervision.

**Daniel Giraldo:** Conceptualization, Methodology, Writing- Reviewing and Editing, Investigation, Project administration.

## Registration of research studies

NA.

## Guarantor

Dora Angélica Corrales Portales.

Daniel Andres Giraldo Benites.

## Provenance and peer review

Not commissioned, externally peer-reviewed.

## Appendix A Supplementary data

Fig. S1 Sequence and order of events in the patient’s attention.
