# Supplementary figures and images for: Intestinal necrosis caused by acute mesenteric ischemia associated with pregnancy: A case report and literature review
Source: Int J Surg Case Rep. 2020 Aug 21;74:164–7. doi: 10.1016/j.ijscr.2020.08.009 (PMC7475227; doi:10.1016/j.ijscr.2020.08.009)

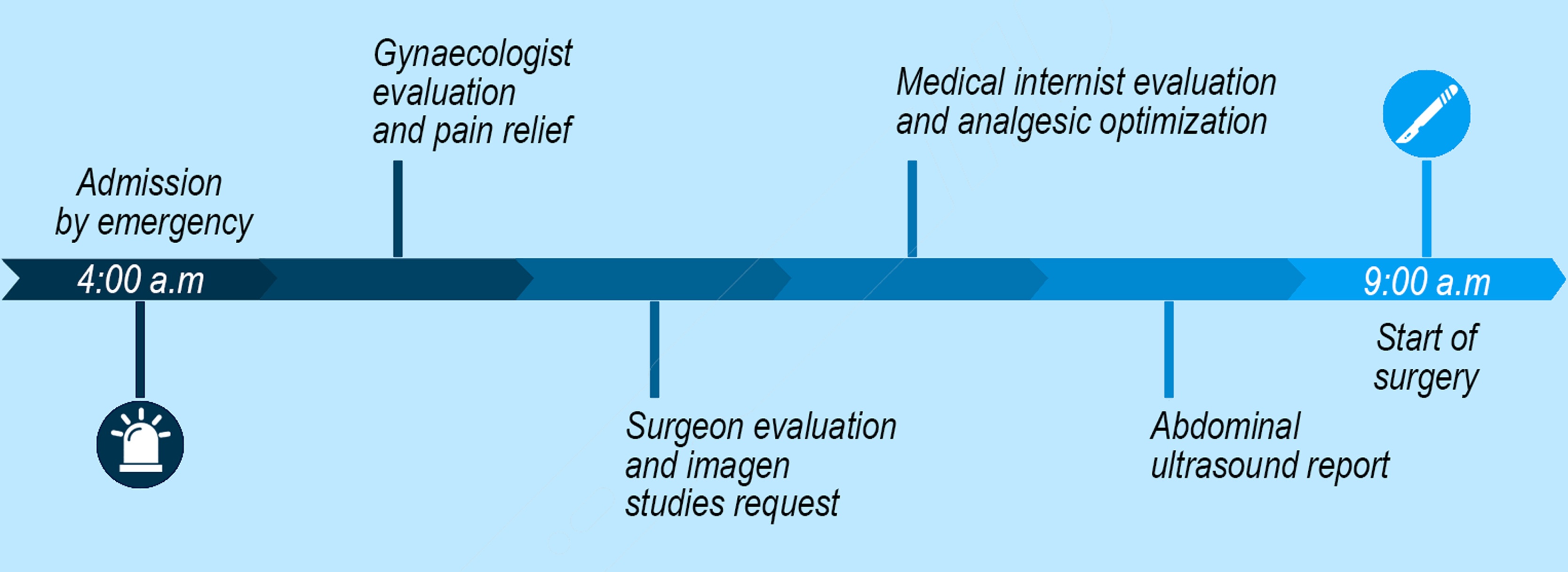

Supplement: Fig. S1 — Sequence and order of events in the patient’s attention. [file mmc1.jpg]
